# Long-Lasting Electret Melt-Blown Nonwoven Functional Filters Made of Organic/Inorganixc Macromolecular Micron Materials: Manufacturing Techniques and Property Evaluations

**DOI:** 10.3390/polym15102306

**Published:** 2023-05-14

**Authors:** Jia-Horng Lin, Yan-Yu Lin, Yang-Min Sue, Mei-Chen Lin, Yueh-Sheng Chen, Ching-Wen Lou

**Affiliations:** 1 College of Material and Chemical Engineering, Minjiang University, Fuzhou 350108, China; jhlin@fcu.edu.tw; 2 Laboratory of Fiber Application and Manufacturing, Advanced Medical Care and Protection Technology Research Center, Department of Fiber and Composite Materials, Feng Chia University, Taichung 407102, Taiwan; happysue311059@gmail.com; 3 School of Chinese Medicine, China Medical University, Taichung 404333, Taiwan; yuehsc@mail.cmu.edu.tw; 4 Advanced Medical Care and Protection Technology Research Center, College of Textile and Clothing, Qingdao University, Qingdao 266071, China; 5 Department of Biomedical Engineering, College of Biomedical Engineering, China Medical University, Taichung 404333, Taiwan; 6 Department of Bioinformatics and Medical Engineering, Asia University, Taichung 413305, Taiwan; 7 Department of Medical Research, China Medical University Hospital, China Medical University, Taichung 404333, Taiwan; 8 Fujian Key Laboratory of Novel Functional Fibers and Materials, Minjiang University, Fuzhou 350108, China

**Keywords:** melt-blown nonwoven, montmorillonite (MMT), titanium dioxide (TiO_2_), carbon nanotube (CNT)

## Abstract

Melt-blown nonwoven fabrics for filtration are usually manufactured using polypropylene, but after a certain time period the middle layer of the mask may have a reduced effect on adsorbing particles and may not be easily stored. Adding electret materials not only increases storage time, but also shows in this study that the addition of electret can improve filtration efficiency. Therefore, this experiment uses a melt-blown method to prepare a nonwoven layer, and adds MMT, CNT, and TiO2 electret materials to it for experiments. Polypropylene (PP) chip, montmorillonite (MMT) and titanium dioxide (TiO_2_) powders, and carbon nanotube (CNT) are blended and made into compound masterbatch pellets using a single-screw extruder. The resulting compound pellets thus contain different combinations of PP, MMT, TiO_2_, and CNT. Next, a hot pressor is used to make the compound chips into a high-poly film, which is then measured with differential scanning calorimetry (DSC) and Fourier transform infrared spectroscopy (FTIR). The optimal parameters are yielded and employed to form the PP/MMT/TiO_2_ nonwoven fabrics and PP/MMT/CNT nonwoven fabrics. The basis weight, thickness, diameter, pore size, fiber covering ratio, air permeability, and tensile property of different nonwoven fabrics are evaluated in order to have the optimal group of PP-based melt-blown nonwoven fabrics. According to the results of DSC and FTIR measurements, PP and MMT, CNT, and TiO_2_ are completely mixed, and the melting temperature (T_m_), crystallization temperature (T_c_) and endotherm area are changed accordingly. The difference in enthalpy of melting changes the crystallization of PP pellets, which in turn changes the fibers. Moreover, the Fourier transform infrared (FTIR) spectroscopy results substantiate that PP pellets are well blended with CNT and MMT, according to the comparisons of characteristic peaks. Finally, the scanning electron microscopy (SEM) observation suggests that with a spinning die temperature of 240 °C and a spinning die pressure lower than 0.01 MPa, the compound pellets can be successfully formed into melt-blown nonwoven fabrics with a 10-micrometer diameter. The proposed melt-blown nonwoven fabrics can be processed with electret to form long-lasting electret melt-blown nonwoven filters.

## 1. Introduction

Air pollution has become a global threat to public health. Epidemiology reports indicate that exposure to air pollution has caused death tolls in the millions [1]. The lungs and related organs are developed in infancy and childhood, which means that infants and young children are vulnerable to environmental pollution and prone to respiratory diseases because of air pollution [2]. Air-borne pollutants can originate from both indoor and outdoor sources in the form of vapor or particles that are composed of different chemicals [3]. Recent research shows that air pollution is putting human respiratory health in jeopardy at an escalating rate. Moreover, the WHO estimates that nine out of every ten people in the world suffer from highly polluted air, and it is estimated that 7 million people have died from air pollution in the world, including 0.66 million children [4]. In 2016 alone, air pollution killed 4.2 million people. The lung functions do not develop completely until adolescence because the majority of alveoli start growing after babies are born, which makes children rather than adults more likely to fall victim to air pollution. By contrast, children tend to have greater contact with air pollution than adults as children are highly energetic and have greater breath volume, and engage in more outdoor activities [5]. Furthermore, the negative influence of air pollution on the respiratory tract starts from a young age and lasts to adulthood, causing an escalating risk of respiratory tract diseases in adults [6,7,8,9,10,11].

Polypropylene (PP) is a semi-crystallized thermoplastic material that is non-toxic, odorless, and ivory-white. It is mainly composed of polymerized single propylene and has a neat molecular structure and a high crystallinity. In addition to its low impact resistance, polypropylene also has a comparable structure of polyethylene, and hence many similar features, such as ease of processing, good chemical stability, and abundance access to raw material [12,13]. In addition, Montmorillonite (MMT) consists of silicate layers of a length of 218 nm and a thickness of 1 nm, and the crystal structure is composed of two silicate tetrahedral layers that seal one center alumina octahedron laminate. In addition, MMT is chemically composed of hydrates, e.g., sodium, calcium, magnesium, aluminum, polysilicates, and hydroxide, so the water content is not specified. As water molecules can easily permeate the loose interlayer structure of MMT, the resulting mineral presents different colors depending on the place of origin. 

The incorporation of electret with nonwoven fabrics can apply to air filters, e.g., masks, strainer net, and alternate materials for filtering devices. Electret provides a stable voltage which is also helpful when made into electronic parts, such as electrical machines, electric igniters, air filters, and high-tension supply. 

## 2. Experimental Section

### 2.1. Materials

Polypropylene pellets (YUNGSOX1080; Formosa Plastics, Taiwan) had a melting index of 10 g/10 min (ISO1133) and a density of 0.9 g/cm^3^. Polypropylene masterbatch (MetoceneMF650Y; Polymirae Co., Ltd., Seoul, Republic of Korea) had a melting index of 1800 g/10 min. Titanium dioxide (TiO_2_) powders (R103; E. Chang Trading Co., Ltd., Taipei, Taiwan, China) had a purity of 98% and a particle size of 0.23 μm. Montmorillonite (MNT) was purchased from Southern Clay Products Co., Ltd., Austin, TX, USA. Carbon nanotubes (CNTs; CF182C; Ming Hsin Co., Ltd., Taiwan, China) had a diameter of 10–30 nm. Commercially available masks (type I) (Shuo Ting Precision Ind. Co., Ltd., Taichung, Taiwan, China) had a specification of W7.5 cm × H19.5 cm and consisted of a waterproof layer, an antibacterial agent layer, and a tender absorbent sheet. Commercially available masks (type II) (Medicom Asia-Pacific Holdings Limited Taiwan Branch, HK, China) had a specification of W9.0 cm × H17.4 cm, an antimicrobial activity ≧95%, and a differential pressure <5.0 (mmH_2_O/cm^2^). 

### 2.2. Preparation of Compound Pellets 

**Figure 1:**
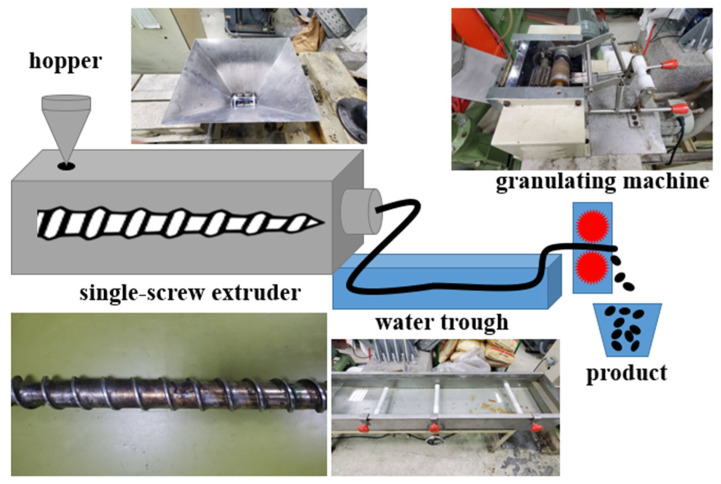
Diagram of melt-blending process.

For the starter, PP chips were used as the staple material and MMT, TiO_2_, and CNT were melt-blended using a single-screw blender. Different melts were drafted into a water trough and with a granulator, PP/MMT/TiO_2_ and PP/MMT/CNT pellets were prepared as in Figure 1. Next, with a hot pressor, the pellets were transformed into high-poly films that were tested with DSC and FTIR to determine the optimal parameters. Both MMT/TiO_2_ ratios and MMT/CNT ratios included 4/0, 3/1, 2/2, 1/3, and 0/4. 

### 2.3. Preparation Melt-Blown Nonwoven Fabrics

**Figure 2:**
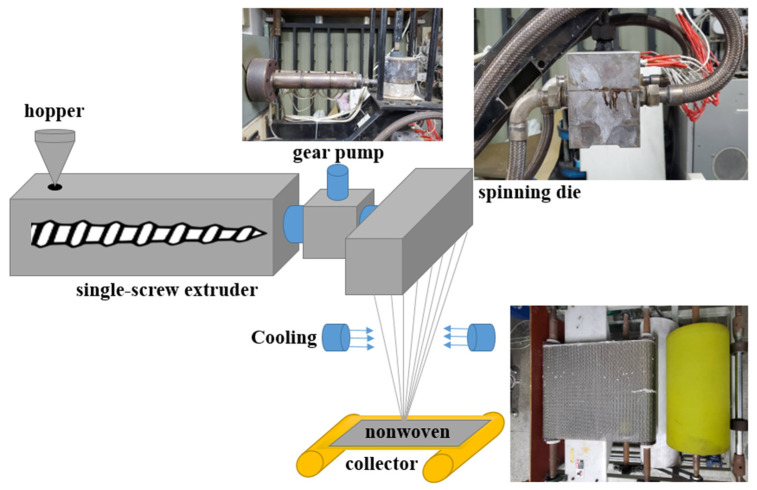
Manufacturing process of melt-blown nonwoven fabrics.

To begin with, the compound pellets were poured into a melt-blown nonwoven machine, and then extruded by a single screw for the pre-mixing. The compound pellets were fully mixed and then drawn into the spinning die by a screw. The three-section temperature of the die was 175, 280, and 300 °C, respectively. In the meanwhile, the temperature was 270 °C for the air pipe, 230 °C for the metering pump, 200 °C for the spinning die, and 180 °C for the blast heater. With a screw extruder pressure of 8~12 MPa, a high-pressure gas compressed the pellets from the spinning die, forming a reticular net that was then fully expanded to form fibers that were eventually coiled over the collector, as shown in Figure 2. The coil rate was adjusted during the process. The melt-blown nonwoven fabrics were observed using an SEM and also measured for thickness, basis weight, diameter, air permeability, and flexibility. The study mainly investigated the manufacturing technique and properties of compound pellets with three types of electret materials being incorporated with the staple material polypropylene, and samples had specifications as shown in Table 1. 

### 2.4. Tensile Test

As specified in ASTMD5035-06 test standard, a universal tester (Hung Ta Instrument Co., Ltd., Taiwan, Taichung, China) was used to measure the tensile strength and elongation at breakage of composites at a test rate of 305 ± 13 mm/min. Rectangle samples had a size of 180 mm × 25.4 mm, and eight samples for each specification were averaged with standard error. 

### 2.5. Scanning Electron Microscopy (SEM)

Composites were affixed to the base with carbon paste and then coated with a thin layer of gold for sixty seconds. An SEM equipped with working voltage of 2 kV and 5 kV was used to observe the cutting section of the composites. 

### 2.6. Differential Scanning Calorimetry (DSC)

The sample weighing 8–10 mg was placed in the sample plate of DSC, and then heated at increments of 10 °C/min from 40 °C to 200 °C where it was kept for five minutes in order to remove the thermal history of the material. Next, the sample was cooled at increments of 10 °C/min until the temperature was 40 °C. The sample was once again heated to 200 °C in increments of 10 °C/min.

### 2.7. Fiber Diameter Analysis

The SEM images were analyzed using imageplus6.0 software. Prior to the measurement, the scale was specified in the software. One hundred counts of fibers were taken from the control group and the experimental groups for comparison. 

### 2.8. Fourier Transform Infrared Spectroscopy (FTIR)

FTIR was used to measure the functional group of the sample at a screen range of 400~4000 cm^−1^. Potassium bromide (KBr) and samples were separately hot-pressed into films. KBr film was scanned to serve as the control group and then compared with the experimental groups. 

### 2.9. Fiber Covering Ratio

SEM images were imported to image pro plus 6.0. The void area in a quantified area was marked and then subtracted from the quantified area. The values were then averaged.

### 2.10. Filter Performance, Pressure Drop, and Quality Factor Tests

An electrical low-pressure impactor (TSI 3321) was used to evaluate the filter performance of nonwoven fabrics. Eight samples for each specification were used for the test. The particle concentrations beyond and beneath the sample were measured and computed with the subsequent equation for filter efficiency:

E = (1 − ηi/Νi) × 100%

where ηi is the particle concentrations beyond the filter sample while Νi is the particle concentrations beneath the filter sample. 

During the filter performance test, a Micromanometer (Models PVM 610, Airflow Measurements Ltd., Bolton, UK) was used to measure and record the pressure drop between two ends of the filter sample.

The quality factor was finally computed with the following equation:

Q = (−ln(1 − E))/∆P

where Q is the quality factor, E is the filter efficiency, and ΔP is the pressure drop.

## 3. Results and Discussion

### 3.1. DSC Analyses of PP/MMT/CNT/TiO_2_ Films

**Figure 3:**
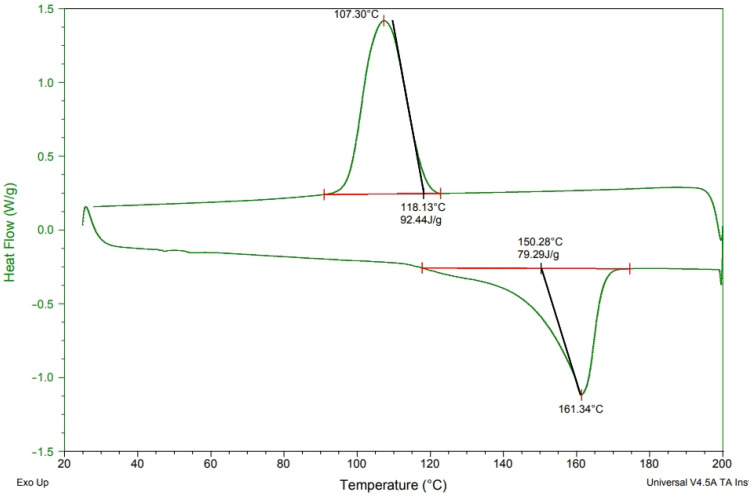
DSC analysis of polypropylene fibers at a heating rate of 10 °C/min.

**Figure 4:**
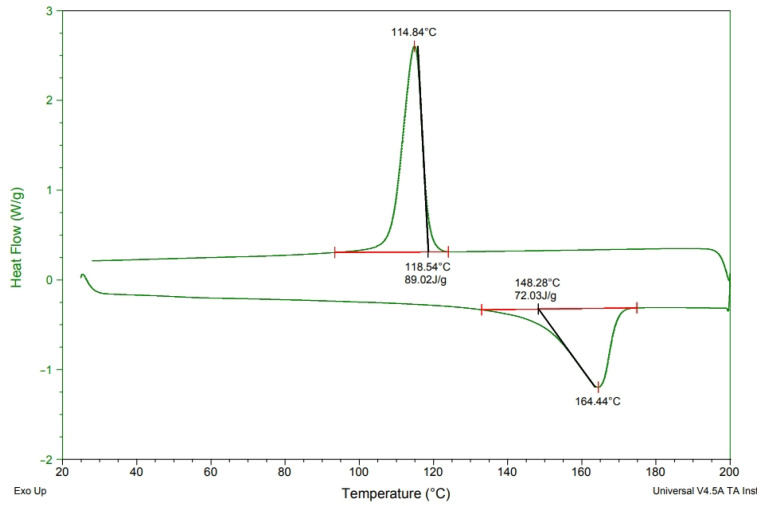
DSC analysis of PP/4M at a heating rate of 10 °C/min.

**Figure 5:**
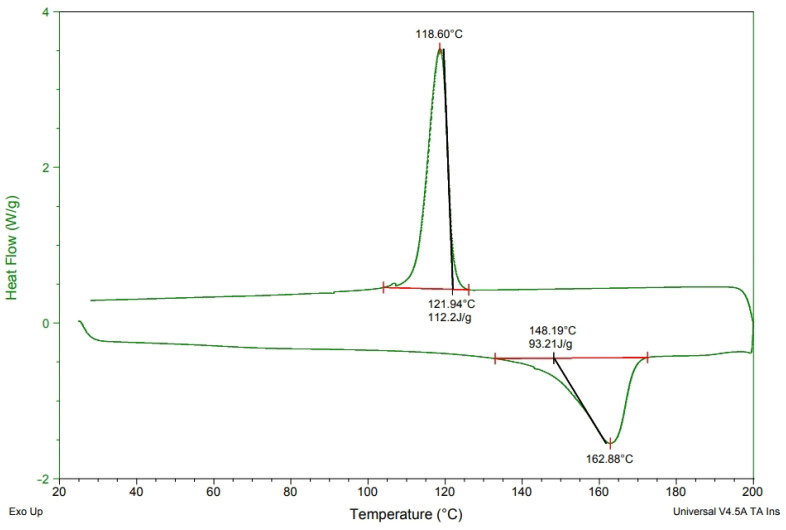
DSC analysis of PP/4T at a heating rate of 10 °C/min.

**Figure 6:**
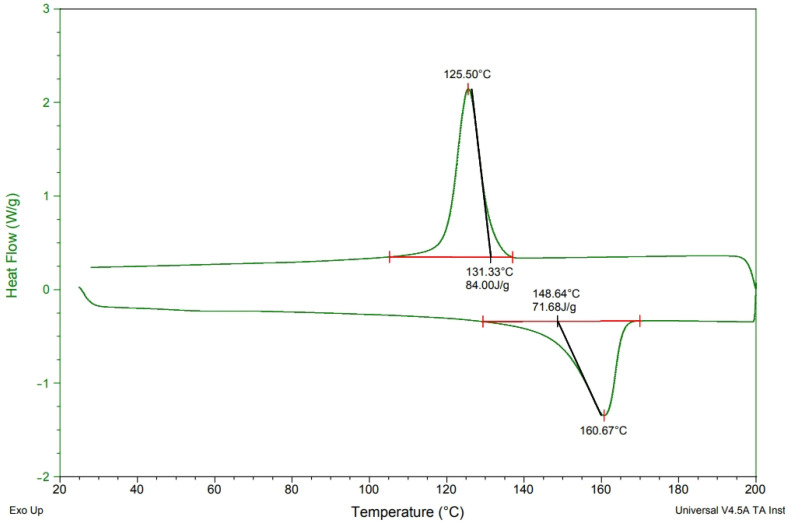
DSC analysis of PP/4C at a heating rate of 10 °C/min.

Figure 3, Figure 4, Figure 5 and Figure 6 show the heat of melting and crystallization of samples when the DSC measurement renders heating at increments of 10 °C/min. The electret includes CNT, MMT, and TiO_2_ that are incorporated with PP pellets at various ratios. The electret-containing compound pellets were measured for melting temperature (T_m_), crystallization temperature (T_c_), melting temperature (T_m_), and endotherm area. There were some differences in the Tc and endotherm area [14], which prove that the presence of electret material renders PP with thermal transformation [14,15]. Subsequently, the crystallinity of PP pellets is changed in response. Table 2 shows the computed crystallinity. On top of the melting enthalpy, the presence of TiO_2_ can also improve the crystallinity of PP. According to the equation, ΔHm is the heating curve of melting enthalpy, wi is the content of electret, and ΔH*f is the 100% theoretical melting enthalpy for PP. After charging, the ability to retain trapped charge inside the melt-blown nonwoven fabrics is significantly dependent on the crystalline region and amorphous region of fibers [14,15,16]. 

Figure 2 summarizes the DSC results collected from Figure 3, Figure 4, Figure 5 and Figure 6, according to which melt-blown nonwoven fabrics are produced with a certain roughness. Unlike the electrostatic spinning high-poly films that can freely transmit electric charges in all directions, melt-blown nonwoven fabrics can efficiently stop or delay the electric charge transport, which in turn slows down the attenuation of electric charges while strengthening the charge storage time. Based on Table 2, either the 4C group or the 4M group is not associated with the crystallinity of PP. It is hard for the large non-polar PP molecules to be embedded between MMT and CNT, and therefore other measures, such as modification, are demanded. To sum up, simply importing MMT and CNT fails to strengthen the crystallinity of PP [16]. 

### 3.2. FTIR Results of MMT/CNT/TiO_2_ Films as Related to the Ratio

**Figure 7:**
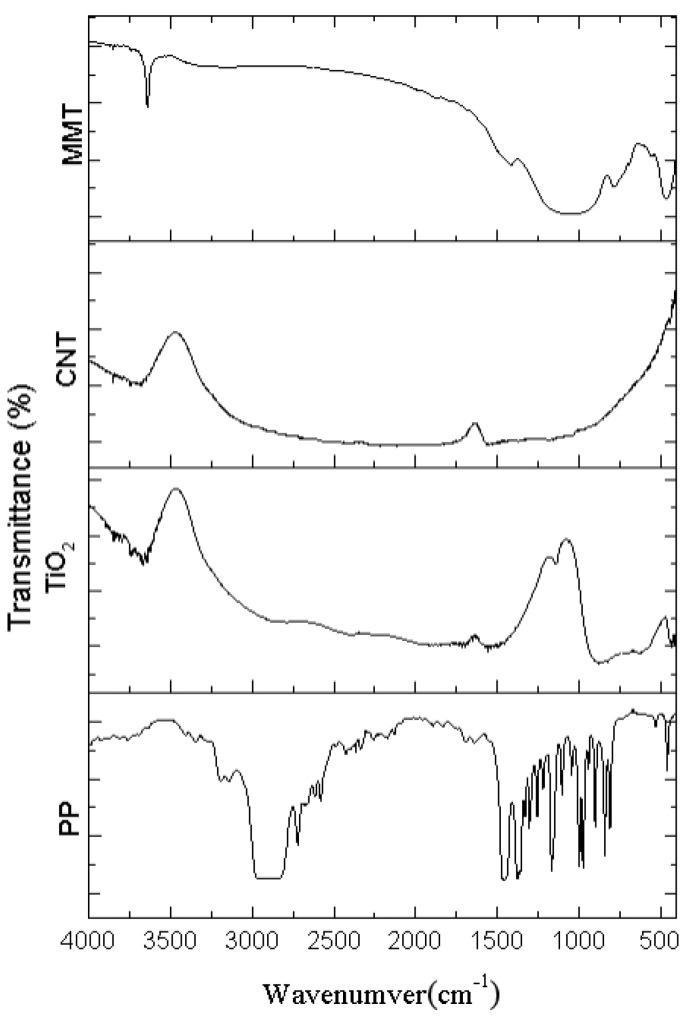
FTIR spectra of PP, TiO_2_, MMT, and CNT.

**Figure 8:**
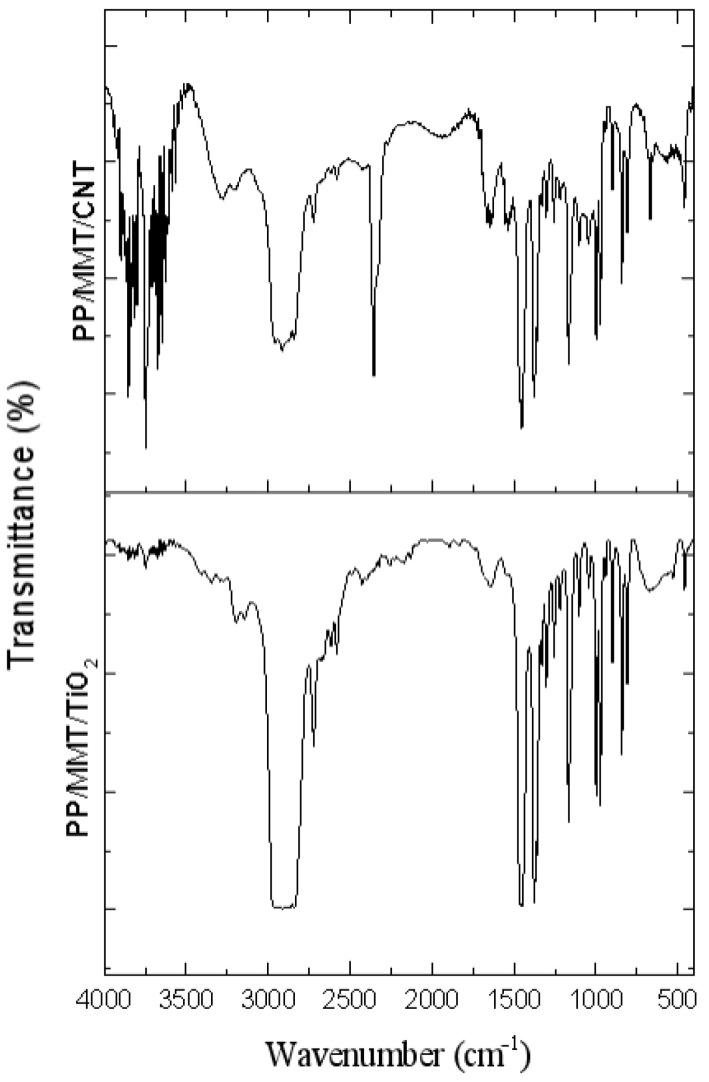
FTIR spectra of MMT/CNT/TiO_2_ melt-blown nonwoven fabrics.

Figure 7 and Figure 8 show the transmittance of the PP pellets and KBr tablets while Table 3 shows the characteristic peaks of samples. The electret-containing material retains the majority of PP’s attributes [17,18,19] and a small proportion of the characteristic peaks of CNT. These results suggest that the electret has been successfully blended with PP pellets, changing the vibration peaks and functional group peaks of PP [20,21]. Lia et al. proposed an element model showing that when a polymer material was electrically charged, the charging atoms were highly agglomerated in the connection between two functional groups [22,23,24].

### 3.3. Properties of MMT/CNT/TiO_2_ Melt-Blown Nonwoven Fabrics as Related to the Ratio

**Figure 9:**
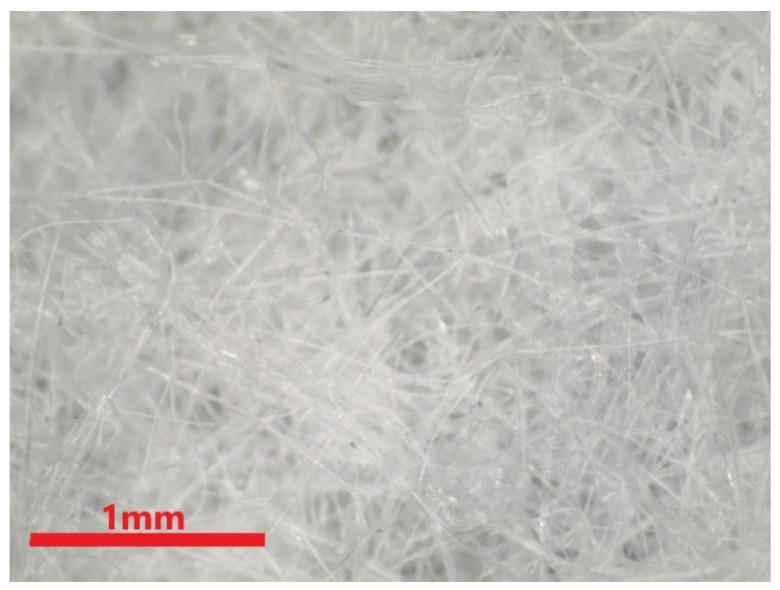
Image of polypropylene melt-blown nonwoven fabrics.

**Figure 10:**
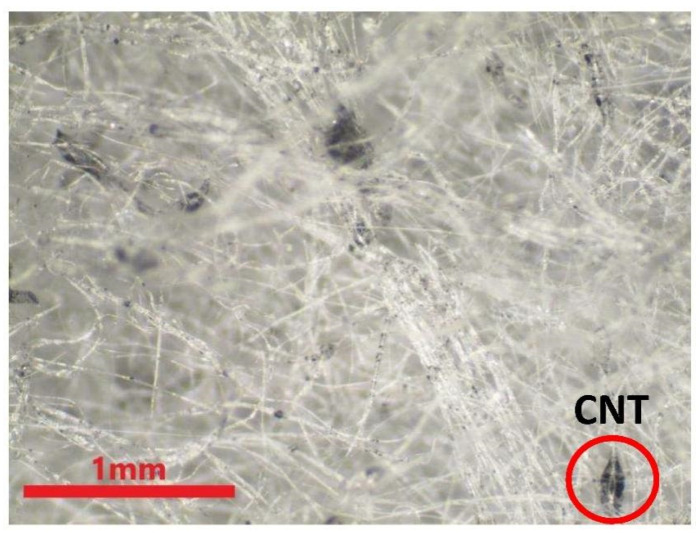
Image of 4M1C melt-blown nonwoven fabrics.

The physical properties of the nonwoven fabrics are examined and discussed in this section. Figure 9 shows the pure PP melt-blown nonwoven net without electret, and the fibers appear to be a darker shade of brown. Additionally, the CNT agglomeration is presented in different fibers [25]. This can improve the agglomeration via modified polypropylene, such as grafting maleic acid anhydride. Wong et al. indicated that CNT commonly adhered to the surface of fibers or polymers. The adhesion of CNT over the fibers is in an irregular shape, as indicated by the red circles in Figure 10, the result of which is in the conformity of the finding of the previous study. To sum up, this study successfully mixes the electret material with PP pellets, and the resulting electret-containing nonwoven fabrics are used in the subsequent discussions. 

### 3.4. Effect of Die Temperature, Air Temperature, and Spinning Distance on Melt-Blown Nonwoven Fiber

**Figure 11:**
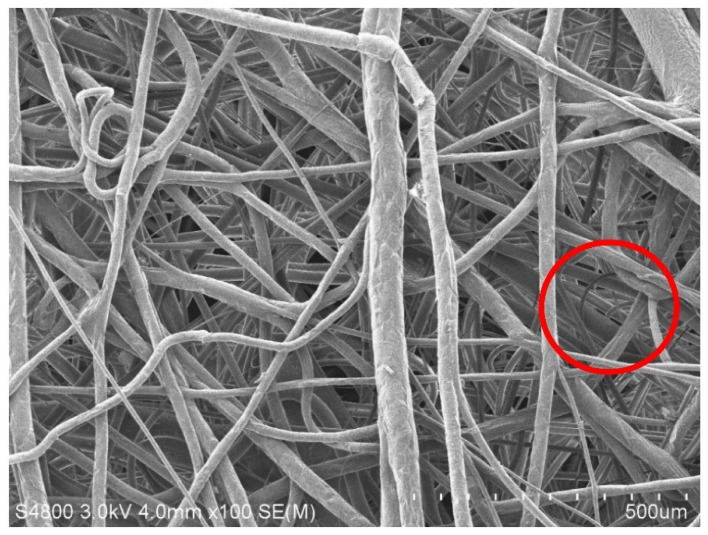
SEM image of polypropylene fibers at 200 °C.

**Figure 12:**
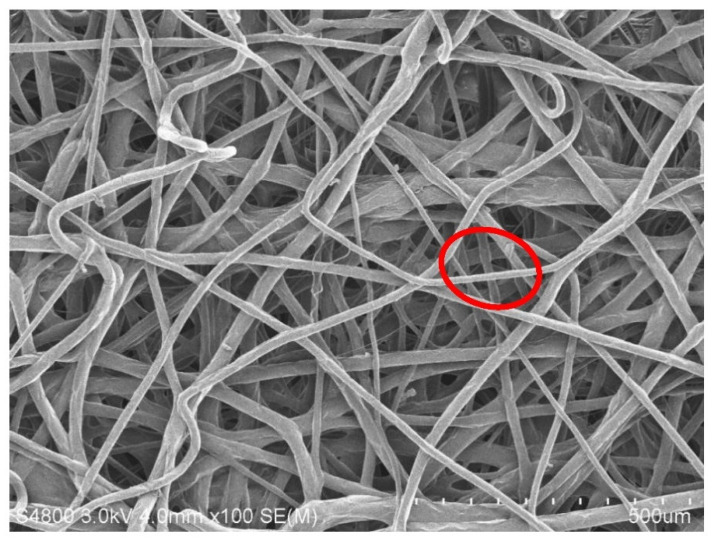
SEM image of polypropylene fibers at 240 °C.

Table 4 shows that with a rise in the temperature of the die, the diameter of fiber gradually decreased, with the PP melt extruding through the die, reaching the collector, and eventually expanded and cooled into fibers. With a specified distance between the die and the collector, increasing the temperature of the die increases the cooling time for fibers deposited over the collector. When constantly increasing the temperature of the die, the resulting fibers become thicker, which is surmised to be a result of thermal accumulation. Moreover, increasing the temperature of the die also causes a compact fiber accumulation, which may be ascribed to the fact that the PP melt is not cooled completely over the collector yet continues to be wrapped in subsequent cooled fibers as in Table 4. The compact accumulation affects the air permeability, thickness, basis weight, and the final filtration of the melt-blown nonwoven fabrics. A rise in the temperature of hot wind does not affect the diameter as much as a rise in the temperature of the die [26]. Figure 11 and Figure 12 show the SEM images of fibers at a magnification of 100 as related to the temperatures of 200 °C and 240 °C. By contrast, fibers tended to have greater thickness at 200 °C, and a greater diameter means a larger pore size of the nonwoven fabrics. Nonwoven fabrics with uneven pore sizes adversely affect the charging electrical field, which prevents charges functioning evenly over the nonwoven fabrics, compromising the electrostatic absorption filtration and particle sedimentation for filtering 1~3 μm particles. 

### 3.5. Effects of Collection Distance and Air Pressure on Melt-Blown Nonwoven Fabrics

According to Table 5, the fiber diameter is dependent on the collection distance and air pressure, and the fiber diameter also changes the air permeability of fibers. When the collection distance increases from 35 cm to 40 cm, it allots a greater time and length for the polymer melt moving through the air, and the fibers can be expanded further to have greater fineness. Moreover, a rise in the air pressure reduces the fiber diameter. When a polymer melt moves through a die under high air pressure to arrive at the collector, the fiber diameter is proven to be reduced [27,28]. 

**Figure 13:**
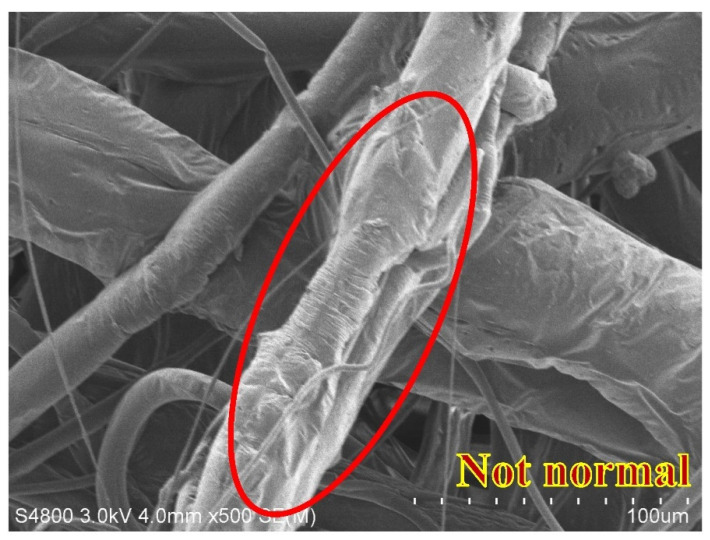
SEM image of polypropylene fibers with a collection distance of 35 cm and an air pressure of 0.008 MPa.

**Figure 14:**
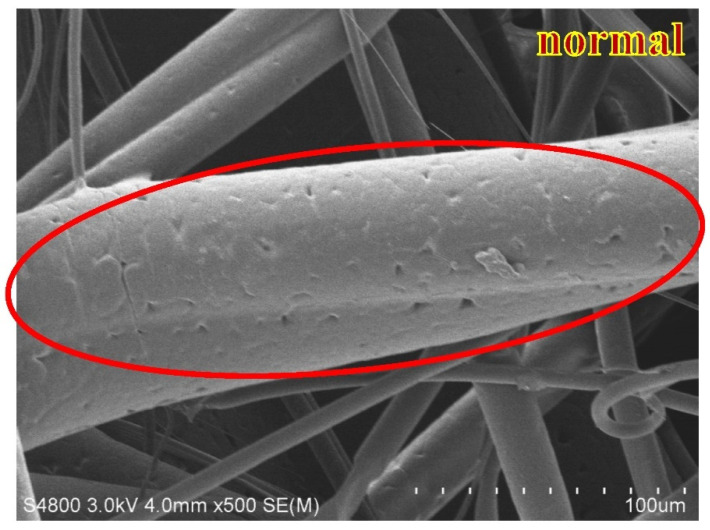
SEM image of polypropylene fibers with a collection distance of 40 cm and an air pressure of 0.010 MPa.

Figure 13 and Figure 14 show the SEM images of melt-blown nonwoven fabrics at a magnification of 500. The images indicate that without fully expanded fibers, the accumulation of fibers over the collector causes a rugged surface of nonwoven fabrics. A greater diameter of fibers may be attributed to the fact that, when extruded through the die by the high-pressure gas, the polymer melt fails to be expanded and cooled into fibers, but pulled by the fore fibers to be collected over the collector. The fibers become curly due to the stretch during the melt-blowing process, which in turn renders an ascending trend in the diameter, unevenness of diameter or an increase in thickness. A uniform charging capacity among fiber surfaces is correlated with the average diameter, and the uneven electrical charges exert a significantly negative influence over the electrostatic adsorption capacity in subsequent filter measurement [29]. Abnormal fibers have a relatively distorted fiber shape, as can be seen in Figure 13. The fiber shape is distorted and uneven, and compared to Figure 14, the fiber is flat and full. Abnormal fibers are usually affected by the manufacturing process, including rapid cooling of the material in the spinning die extrusion and cooling portions, resulting in temperature differences in the inner and outer structures of the fibers; additionally, the extrusion result caused by the nonwoven fabric after the collection device material is collected by the roller.

### 3.6. Effect of Collector Speed on Melt-Blown Nonwoven Fibers 

Table 6 shows that the thickness and basis weight of melt-blown nonwoven fabrics is correlated with the rotary speed of the collector. The tests were conducted with various rotary speeds of the collector, but with a specified die temperature of 240 °C, a specified air temperature of 180 °C, a specified pressure of 0.010 MPa, and a specified collection distance of 40 cm. When the roller of the collector operates at a lower rate, both the thickness and basis weight of fibers are increased. A certain thickness of fibers adversely affects the air permeability as well as the subsequent pressure drop measurement. The melt-blowing process involves a polymer melt being blown by a high-pressure gas and then fully expanded while being completely cooled into straight fibers before they fall onto the rotary collector. If the process fails in these steps, fibers are made with uneven diameters [30,31]. 

### 3.7. Effect of Fiber Diameter and Covering Ratio on Melt-Blown Nonwoven Fabrics

The fiber diameter is correlated with the pore size and fiber covering ratio. Similarly, pore size is correlated with the air permeability and filtration performance [26,31]. Therefore, spinning die temperature and air pressure can be adjusted to allow fibers to be expanded and cooled to a certain level, attaining the minimal diameter of 10μm that competes the fiber diameter of commercial masks in order to proceed the subsequent filtration performance measurement for further comparisons [32].

### 3.8. Tensile Performance of Melt-Blown Nonwoven Fabrics as Related to the Ratio 

**Figure 15:**
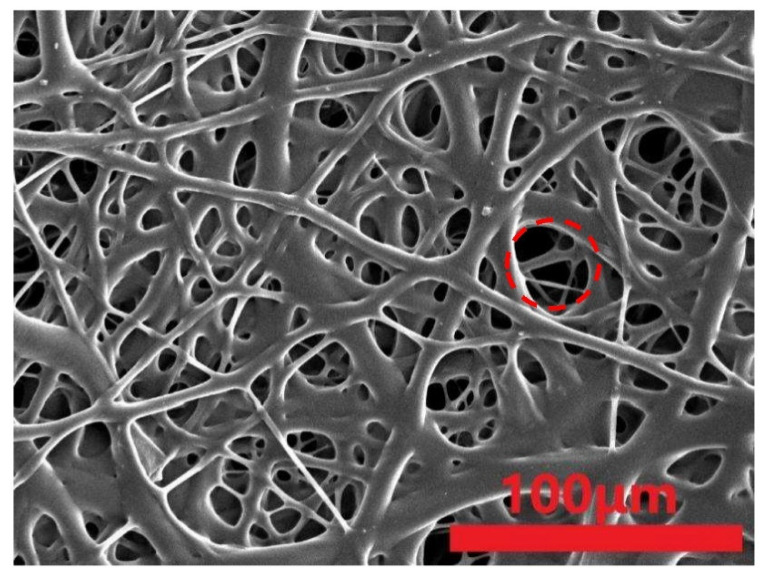
SEM image of commercial mask (500×).

**Figure 16:**
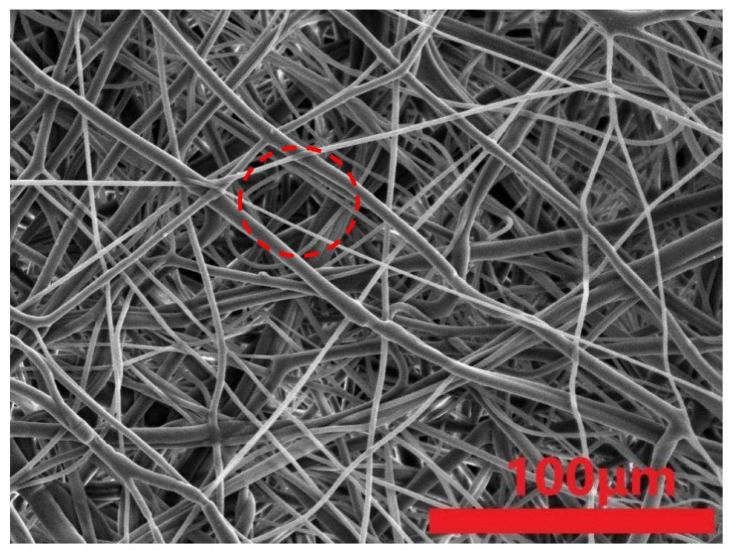
SEM image of melt-blown nonwoven fabrics containing 4 wt% TiO_2_ as electret.

**Figure 17:**
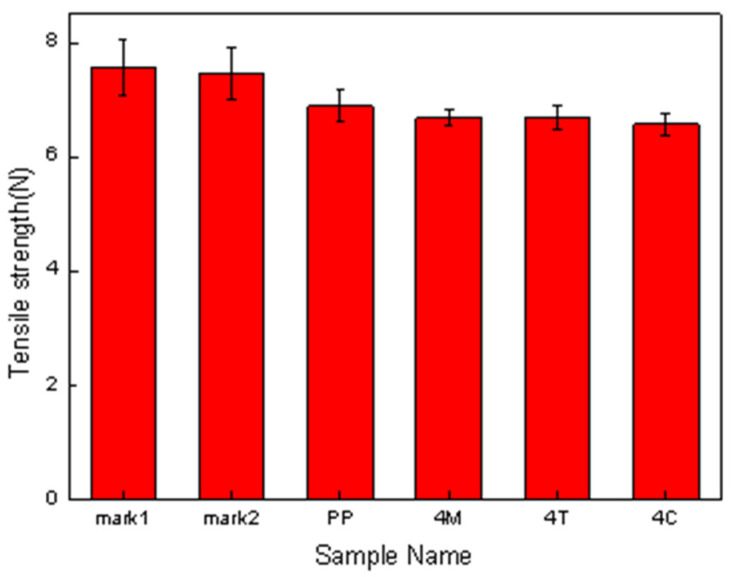
Tensile strength of melt-blown nonwoven fabrics containing PP, 4 wt% of MMT, 4 wt% of TiO_2_, and 4 wt% of CNT.

Comparing Figure 15 and Figure 16, the commercial masks demonstrate a greater fiber covering ratio than the proposed melt-blown nonwoven fabrics, which is surmised to be attributed to the manufacturing parameters referring to tables regarding physical properties. Additionally, this is also correlated with the pore size of the spinning die equipped with the melt-blowing machine [33,34,35]. Figure 17 shows that the tensile performances of fibers are in direct proportion to the fiber covering ratio. Namely, the tensile strength is correlated with fibers in terms of average pore size and fiber covering ratio. Table 7 shows that an increase in the fiber covering area means that fibers can disperse a force evenly in directions when being exerted with an external force. This phenomenon also means that the stress can be better dispersed to the fiber net. 

### 3.9. Air Permeability of Melt-Blown Nonwoven Fabric as Related to the Ratio of Materials 

**Figure 18:**
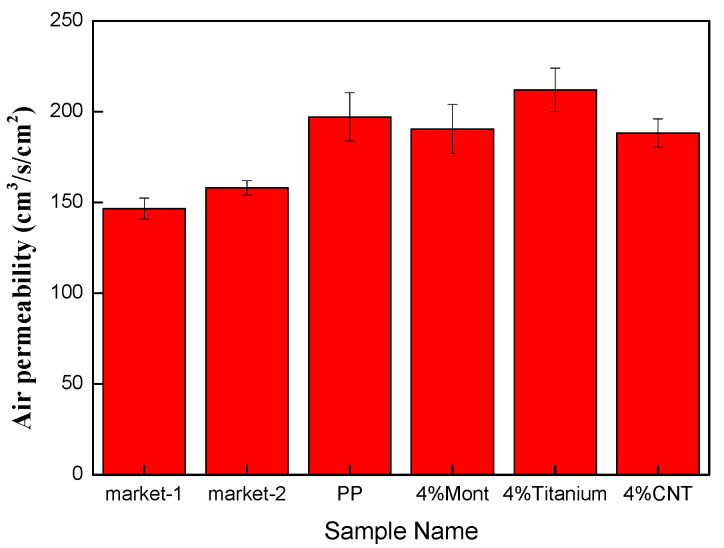
Air permeability of melt-blown nonwoven fabric containing PP, 4 wt% of MMT, 4 wt% of TiO_2_, and 4 wt% of CNT.

Figure 18 shows that the air permeability is highly pertinent to the pore size and fiber diameter (c.f. Table 7). The control groups, and commercial mask type I and type II, show an average pore size that is correlated with fiber diameter and fiber covering ratio. With a smaller pore size, the air permeability has a descending trend, which means that the fiber net exhibits a lower air inflow and a higher pressure drop [36,37]. Supported by the SEM images, the control groups demonstrate a smaller pore size and a larger fiber covering ratio. In comparison, the air permeability of the experimental group is approximately 220 cm^3^/s/cm^2^, which benefits the air inflow of the fiber nets while reducing the pressure drop on both side of the fiber nets [38,39].

### 3.10. Pressure Drop of Nonwoven Fabrics 

**Figure 19:**
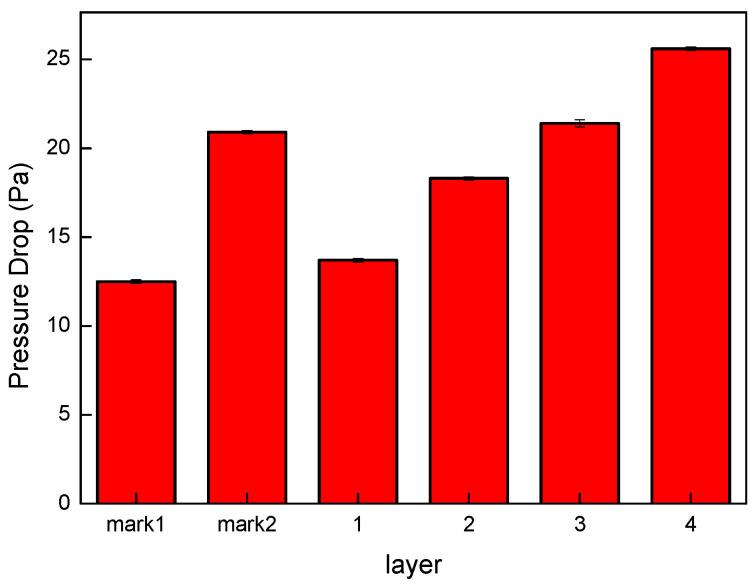
Pressure drop of melt-blown nonwoven fabrics.

As per regulation in Taiwan, medical masks have to pass the pressure drop test as specified CNS14777, the pressure drop is required to be smaller than 5 mmH_2_O/cm^2^, meaning a pressure of 48.9 Pa. Figure 19 shows the pressure drop of three-layered commercial masks as well as the pressure drop of melt-blown nonwoven fabrics as related to the number of lamination layers. With a number of lamination layers between 1 and 4, the melt-blown nonwoven fabrics fail to reach 48.9 Pa, so they are qualified for the medium filter layer exclusively. The increment in the pressure drop is correlated with the pore size, thickness, and fiber coverage of the nonwoven fabrics. A rise in the number of lamination layers increases the pressure drop of melt-blown nonwoven fabrics (Figure 19). 

### 3.11. Filter Performance of Nonwoven Fabrics 

The pore size of nonwoven fabrics is the voids among the constituent fibers, and the pores can filter the particles passing through the nonwoven fabrics with five mechanisms, including interception, inertia, random diffusion, electrostatic adhesion, and gravity action. The mechanisms work with corresponding particle sizes. Particles with a size smaller than 0.3 μm are mainly blocked by diffusion and electrostatic adhesion mechanisms. Particles with a size between 0.3~1 μm are mainly blocked by the interception mechanism. The inertia mechanism and the gravity action mechanism are used for blocking particles of 1~10 μm and greater than 10 μm, respectively. Figure 19 and Table 8 show that single-layer nonwoven fabrics possess lower pressure drop and an excellent filter efficiency, which will facilitate the final application of using nonwoven fabrics as filter material. Moreover, it is also proven that a combination of a certain amount of electret with polypropylene is beneficial to improving filter efficiency. The filter efficiency is improved from 13% to 33%, suggesting that the filter efficiency of nonwoven fabrics is highly dependent on the presence of electret. 

### 3.12. Quality Factor of Nonwoven Fabrics

Quality factor is an important evaluation criterion when filter performance of nonwoven fabrics is concerned. Because of the thickness, basis weight, and other vicarious factors of the constituent materials of nonwoven fabrics, it is difficult to judge the filter efficiency of the filters directly, which makes the computation of quality factor indispensable. Table 9 shows that nonwoven fabrics that are incorporated with electret have an improved quality factor that is increased from 0.08 Pa^−1^ to 0.12 Pa^−1^. The quality factor is computed with the equation Q = (−ln(1 − E))/∆P, where Q is the quality factor, E is the filter efficiency, and ΔP is the pressure drop. It can be expected that the commercially available three-layered masks (outer, medium, and inner layers) outperform the a single-layer melt-blown nonwoven fabric in terms of filter efficiency. A three-layered lamination certain demonstrates better filter efficiency against particles at a size of 1μm. Because the filter efficiency against 0.3~1 μm particles is dependent on thickness and pore size, it makes sense that single-layer melt-blown nonwoven fabric shows inadequate performance compared to the commercial masks. However, Figure 9 indicates that the presence of electret has a positive influence on the filter efficiency; namely, electret-containing melt-blown nonwoven fabrics demonstrate good filter performance.

## 4. Conclusions

In this study, a single-screw extruder was employed to melt-blend PP, MMT, TiO_2_, and CNT at different ratios into polymer compound pellets. The pellets were formed into a reticular net using a melt-blown nonwoven machine. According to the results of DSC and FTIR, PP was totally blended with MMT, CNT and TiO_2_, and the Tm, Tc, and endotherm area were changed accordingly. The crystallinity of PP pellets is dependent on the melting enthalpy, which in turn causes differences in the fibers. Moreover, FTIR analysis indicates that CNT and MMT can be well blended with the pellets, as substantiated by the comparison of characteristic peaks. The SEM observation suggests that the compound pellets can be transformed into nonwoven fabrics of constituent 10 μm diameter fibers via the melt-blowing process when the temperature of the die is 240 °C and the pressure is 0.01 MP. The yielded melt-blown nonwoven fabrics are qualified for long-lasting electret filters, demonstrating a tensile strength of 7~8 N as well as an air permeability of 200~250 cm^3^/s/cm^2^ that is higher than that of fibers from commercial masks. In the pressure drop test, the pressure of nonwoven fabrics did not exceed 48.9 Pa of the regulatory standard; in the filtration test, the filtration efficiency of polypropylene nonwoven fabrics with electret was improved by 13% to 33% compared to that without electret. In the quality factor test, the addition of electret could increase the quality factor of nonwoven fabrics. In summary, experimental nonwoven fabrics are expected to be used in air filtration projects.

## Figures and Tables

**Table 1:** Specifications of samples.

Name	MMT Temperature (Orillonite) (wt%)	TiO_2_ (wt%)
4M	4	0
3M1T	3	1
2M2T	2	2
1M3T	1	3
4T	0	4
**Name**	**MMT Temperature (Orillonite) (wt%)**	**CNT (wt%)**
3M1C	3	1
2M2C	2	2
1M3C	1	3
4C	0	4

**Table 2:** DSC value as related to the content of components.

Sample Name	T_m_ (°C)	T_c_ (°C)	T_m_ Area (J/g)	T_c_ Area (J/g)	Crystallinity (%)
PP	150.28	107.30	79.29	92.44	29.7
4C	160.67	125.50	71.68	84.00	27.7
4M	164.44	114.84	72.03	89.02	27.9
4T	162.88	118.60	93.21	112.2	36.1

**Table 3:** FTIR characteristic peaks as related to the blending ratio.

Wavenumber (cm^−1^)	Vibration	Functional Group
2950	asymmetrical stretching	CH_3_
2925	asymmetrical stretching	CH_2_
2870	stretching	CH_3_
1456	symmetrical bending	CH_3_
1375	symmetrical bending	CH
1160	wagging	C-H
1160	rocking	CH_3_
998	rocking	CH_3_
996	stretching	C-C
840	rocking	CH_3_
839	rocking	C-H
809	stretching	C-C

**Table 4:** Effect of dye temperature, air temperature, and spinning distance on melt-blown nonwoven fabric.

Die Temperature (°C)	Air Temperature (°C)	Diameter (μm)	Air Permeability (cm^3^/s/cm^2^)	Thickness (mm)	Basis Weight (g/m^2^)
200	160	41.1 ± 4.49	195.7 ± 13.3	1.83 ± 0.05	181 ± 5.1
200	200	38.9 ± 5.13	212.3 ± 13.5	1.95 ± 0.04	183 ± 6.6
220	160	36.8 ± 5.57	189.7 ± 14.6	1.98 ± 0.04	185 ± 7.2
220	200	35.9 ± 3.25	188.5 ± 7.8	1.82 ± 0.02	189 ± 6.9
240	160	40.5 ± 4.23	197.1 ± 6.5	1.88 ± 0.03	186 ± 4.4
240	200	38.3 ± 5.09	190.5 ± 7.4	1.95 ± 0.03	191 ± 7.3

**Table 5:** Effects of collection distance and air pressure on melt-blown nonwoven fabric.

DCD (cm)	Air Pressure (MPa)	Diameter (μm)	Air Permeability (cm^3^/s/cm^2^)	Thickness (mm)	Basis Weight (g/m^2^)
35	0.008	21.1 ± 4.22	212.7 ± 12.4	1.85 ± 0.03	191 ± 3.1
40	0.008	18.9 ± 3.76	187.3 ± 11.2	1.85 ± 0.04	190 ± 4.2
35	0.008	21.2 ± 4.31	213.7 ± 11.9	1.78 ± 0.02	192 ± 5.1
35	0.010	17.3 ± 2.97	171.5 ± 10.1	1.76 ± 0.02	190 ± 2.3
40	0.008	17.5 ± 3.18	197.1 ± 12.3	1.88 ± 0.03	193 ± 3.4
40	0.010	15.1 ± 1.91	190.5 ± 13.7	1.85 ± 0.04	192 ± 4.3

**Table 6:** Effect of collector speed on melt-blown nonwoven fabric.

Collector Speed (m/min)	Diameter (μm)	Air Permeability (cm^3^/s/cm^2^)	Thickness (mm)	Basis Weight (g/m^2^)
1	21.1 ± 4.22	212.7 ± 12.4	1.85 ± 0.03	191 ± 3.1
2	18.9 ± 3.76	187.3 ± 11.2	1.54 ± 0.08	153 ± 7.5
3	21.2 ± 4.31	213.7 ± 11.9	1.23 ± 0.05	102 ± 9.8
4	17.3 ± 2.97	228.3 ± 10.1	0.76 ± 0.04	73 ± 6.9

**Table 7:** Diameter, pore size, and fiber covering ratio.

Sample Name	Diameter (μm)	Pore Size (μm^2^)	Fiber Covering Ratio (%)	Basis Weight (g/m^2^)
Pure PP	9.1 ± 3.07	712 ± 25.3	68.1	183 ± 3.6
4T	9.7 ± 2.34	649 ± 35.7	69.3	191 ± 2.3
4M	8.9 ± 1.94	697 ± 49.3	67.7	188 ± 5.1
4C	9.6 ± 2.63	714 ± 30.7	69.3	199 ± 3.1
Mark1	7.6 ± 1.03	453 ± 25.7	85.6	81 ± 0.9

**Table 8:** Filter efficiency of nonwoven fabrics.

Name	PP (wt%)	TiO_2_ (wt%)	CNT (wt%)	MMT (wt%)	Filtration Efficiency (%)
PP	100	0	0	0	65.5 ± 5.1
4T	96	4	0	0	87.4 ± 3.0
4C	96	0	4	0	83.1 ± 4.1
4M	96	0	0	4	82.3 ± 3.9
3T1C	96	3	1	0	80.6 ± 5.1
3T1M	96	3	0	1	81.2 ± 5.4
1T3M	96	1	3	0	75.3 ± 7.0
3C1M	96	0	3	1	77.6 ± 7.2
1T3M	96	1	0	3	78.5 ± 5.6
1C3M	96	0	1	3	77.3 ± 6.0
2T2C	96	2	2	0	78.2 ± 5.3
2T2M	96	2	0	2	80.4 ± 4.1
2C2M	96	0	2	2	81.2 ± 4.9
Mark1					85.1 ± 3.1
Mark2					90.5 ± 1.5

**Table 9:** Quality factor of nonwoven fabrics.

Name	PP (wt%)	TiO_2_ (wt%)	CNT (wt%)	MMT (wt%)	Quality Factor (Pa^−1^)
PP	100	0	0	0	0.080
4T	96	4	0	0	0.156
4C	96	0	4	0	0.136
4M	96	0	0	4	0.132
3T1C	96	3	1	0	0.123
3T1M	96	3	0	1	0.127
1T3M	96	1	3	0	0.106
3C1M	96	0	3	1	0.113
1T3M	96	1	0	3	0.116
1C3M	96	0	1	3	0.113
2T2C	96	2	2	0	0.116
2T2M	96	2	0	2	0.123
2C2M	96	0	2	2	0.127
Mark1					0.145
Mark2					0.136

## Data Availability

The data presented in this study are available on request from the corresponding author.
